# Diverse characteristics of the urinary excretion of amino acids in humans and the use of amino acid supplementation to reduce fatigue and sub-health in adults

**DOI:** 10.1186/s12937-017-0240-y

**Published:** 2017-03-23

**Authors:** R. H. Dunstan, D. L. Sparkes, M. M. Macdonald, X. Janse De Jonge, B. J. Dascombe, J. Gottfries, C.-G. Gottfries, T. K. Roberts

**Affiliations:** 1 School of Environmental and Life Sciences, University of Newcastle, Callaghan, Australia; 2 School of Environmental and Life Sciences, University of Newcastle, Ourimbah, Australia; 3 Latrobe University, Melbourne, Australia; 4 Department of Chemistry and Molecular Biology, University of Gothenburg, Gothenburg, Sweden; 5 Department of Neuroscience, University of Gothenburg, Gothenburg, Sweden

**Keywords:** Sub-health, Fatigue, Amino acids, Nutritional supplement, Collagen turnover, Urine

## Abstract

**Background:**

The excretion of amino acids in urine represents an important avenue for the loss of key nutrients. Some amino acids such as glycine and histidine are lost in higher abundance than others. These two amino acids perform important physiological functions and are required for the synthesis of key proteins such as haemoglobin and collagen.

**Methods:**

Stage 1 of this study involved healthy subjects (*n* = 151) who provided first of the morning urine samples and completed symptom questionnaires. Urine was analysed for amino acid composition by gas chromatography. Stage 2 involved a subset of the initial cohort (*n* = 37) who completed a 30 day trial of an amino acid supplement and subsequent symptom profile evaluation.

**Results:**

Analyses of urinary amino acid profiles revealed that three groups could be objectively defined from the 151 participants using k-means clustering. The amino acid profiles were significantly different between each of the clusters (Wilks’ Lambda = 0.13, *p* < 0.0001). Cluster 1 had the highest loss of amino acids with histidine being the most abundant component. Cluster 2 had glycine present as the most abundant urinary amino acid and cluster 3 had equivalent abundances of glycine and histidine. Strong associations were observed between urinary proline concentrations and fatigue/pain scores (*r* = .56 to .83) for females in cluster 1, with several other differential sets of associations observed for the other clusters.

**Conclusions:**

Different phenotypic subsets exist in the population based on amino acid excretion characteristics found in urine. Provision of the supplement resulted in significant improvements in reported fatigue and sleep for 81% of the trial cohort with all females reporting improvements in fatigue.

**Trial registration:**

The study was registered on the 18th April 2011 with the Australian New Zealand Clinical Trials Registry (ACTRN12611000403932).

## Background

The amino acids derived from ingested proteins are used as substrates for the biosynthesis of structural and functional proteins in the body. These digested amino acids can be used for oxidative phosphorylation and gluconeogenesis [1, 2] as well as the facilitation of numerous physiological functions including acting as precursors for the biosynthesis of neurotransmitters [3], hormones, phosphoglycerols, glycolipids and nucleic acids [4, 5]. It has been estimated that the skeletal muscles in the human body comprise 40–60% of body mass and thus represent the major repository of the body’s protein [6]. The body does not maintain specific protein stores, but when it is not possible to obtain sufficient protein via ingestion during exercise, ill health or trauma, the body accesses amino acids via the process of proteolysis where the non-myofibrillar proteins display a high turnover rate to meet demands [7]. The amino acids released from this turnover of protein can enter the blood circulation for metabolism as required and can also be lost in sweat and urine [1, 2]. Healthy resting human adults can achieve nitrogen balance by taking in around 0.8 g/Kg/day of protein [6]; humans synthesise approximately 3 g/Kg/day of new protein [8], indicating that reutilisation of amino acids released during protein turnover is critical to health.

High levels of exercise activity would increase the requirement for protein intake and for protein turnover in order to offset the increased losses of free amino acids arising from demands for energy metabolism, tissue repair and recovery processes [8, 9] as well as excretory losses via sweating [10, 11]. Similar increased demands for amino acids would be seen during illness and recovery from trauma. Increased losses of amino acids were observed in breast cancer patients who suffered fatigue during and following radiation therapy, with evidence for altered amino acid homeostasis [12]. Significantly depleted amino acid levels have also been associated with long-term fatigue [13] leading to the proposal that the nitrogen balance in these patients was less than optimal. A negative nitrogen balance may arise if the supply of amino acids is inadequate as a result of insufficient protein ingestion or impaired digestion such that the body’s requirements for protein synthesis cannot be met. The demand for amino acids is met via proteolysis of non-myofibrillar muscle proteins. A prolonged state of negative nitrogen balance could result in proteolysis of myofibrillar proteins which would in turn lead to muscle wasting and damage. Increased metabolic activities to support exercise, mount host defences against infection, or support recovery from illness and injury, place additional demands on protein turnover within the body [6–8].

It has been shown that the levels of several key amino acids including serine, glycine, histidine, alanine and ornithine are present in sweat at much higher concentrations than occur in the plasma [10, 11, 14, 15]. It was proposed that these levels of amino acids in sweat, as has been found to be the case with sweat electrolytes [16], could be achieved by a process of leaching of the amino acids from the natural moisturising factor in the stratum corneum of the skin to combine with the quantities excreted in sweat [10, 11, 15]. The majority of amino acids which constitute the natural moisturising factor are thought to be derived from the protein filaggrin which is primarily composed of glutamine/glutamic acid, arginine/ornithine, serine, proline, glycine, histidine, and aspartic acid/alanine [17]. Loss of amino acids via sweating as a result of exercise, living in a hot climate, or night sweats associated with illness could exacerbate the requirement for proteolysis when the body cannot obtain proteins via ingestion. It is proposed that subjects experiencing a net negative nitrogen balance with depleted amino acid resources would experience fatigue and pain [1, 18, 19]. If a net negative nitrogen balance is associated with fatigue then it would follow that amino acid supplementation would have the potential to assist in restoring nitrogen balance. Provision of free amino acids removes the requirement for digestion of proteins; amino acids can be directly absorbed into the circulation making them rapidly available to the body’s tissues and organs including striated muscle. The development of an amino acid supplement is not straightforward as considerations of solubility, palatability, composition and dosage need to be addressed [20]. A pilot study on healthy male subjects from the general population experiencing non-debilitating fatigue, demonstrated that a 30 day period of daily supplementation with a complex formulation of amino acids resulted in significantly improved scores for fatigue as well as associated alterations in urinary excretion of amino acids [21].

An early study from our laboratory revealed that amino acid excretion profiles could delineate human subjects suffering fatigue into population subgroups [22]. The basis for differentiation was thought to involve genetic differences in kidney reabsorption mechanisms, metabolic efficiencies, skin moisturisation and sweating processes as well as environmental factors such as dietary influences and infective history. The proposal that different human phenotypes exist on the basis of amino acid homeostasis may explain why some subjects are more susceptible to developing chronic fatigue or are better able to cope with different types of exercise challenge. Fatigue illness is highly prevalent in Western society and significant fatigue is a common feature of many medical conditions [23]. Sub-health is a state that falls between health and disease and involves psychological, physical and medical components believed to increase the risk of illness [24].

## Methods

The current study aimed to determine whether healthy individuals could be separated into discrete subgroups based on the makeup of their urinary excretion profiles. Analyses of a broad range of symptoms were assessed to build a symptom index evaluation of general fatigue, general body pains, gastrointestinal function, sleep and vitality for evaluating sub-health in the general healthy population. Following the initial recruitment and urine analysis, healthy subjects that reported non-debilitating levels of fatigue were provided with a complex amino acid supplement to assess whether supplementation for 30 days would alleviate fatigue. Memberships of population subgroups based on amino acid excretion profiles were tested to determine whether subgroup membership influenced the efficacy of the supplement.

**Fig. 1:**
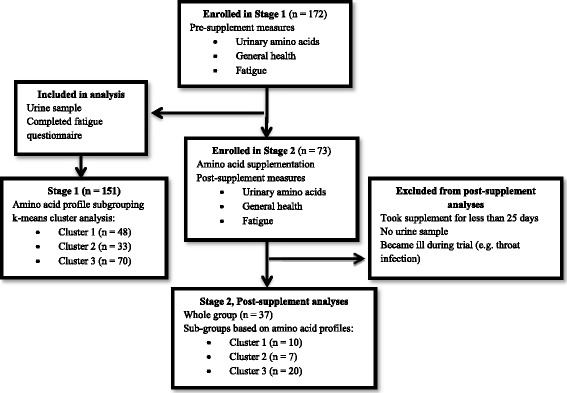
Study design including statistical analyses for the amino acid supplement trial

The study involved recruiting healthy adults who were reportedly free from any significant medical or psychological condition. The project was divided into two stages; 172 subjects were initially recruited to supply urine samples for analyses and complete general health and fatigue questionnaires as summarised in Fig. 1. Recruits were not included if they did not provide urinary samples or they did not complete the questionnaires for evaluation of general health symptoms and fatigue. The standardised timing of the urine sample collection was aimed at assessing the profile composition of amino acids representing homeostasis without contributions of the dietary intake associated with the 24 h collection of urine [25]. Participants therefore provided a fasted first of the morning urine sample which was transported in a Urine-Monovette® 10 ml boric acid tube (Sarstedt, Germany). Amino acid analysis of the urine samples was performed using the commercial EZ: Faast™ derivatisation method (esterification of amino acids) followed by gas chromatography/flame ionisation detection (GC/FID) as previously described [25].

The Chalder fatigue scale questionnaire (total 11 items, seven items related to physical symptoms of fatigue and four items related to mental symptoms of fatigue, Likert scored) was used to assess levels of fatigue; the scale has been validated for use in both a healthy population free from any known medical conditions as well as patients with various forms of chronic fatigue [26]. The current general health of the participants was also assessed using an 86 item questionnaire which was based on a questionnaire previously used by our research team [27–29] which included symptoms covering fatigue, pain, gastrointestinal (GIT), cognitive, neurological and infection related symptoms. Participants were required to report how much they had been affected by these symptoms during the previous 7 days. The questionnaire was Likert scored with responses ranging from 0 “not at all” to 4 “extremely”. Five symptom indices were compiled from symptoms pertaining to the categories of general fatigue, general body pain, GIT function, sleep and vitality in order to evaluate the potential influence of supplementation on general health as shown in Table 1. General body pain and GIT symptoms were monitored to determine whether any adverse side effects were associated with the amino acid supplement. Correlation and factor analyses were carried out to select the minimum number of optimal symptoms required to define each symptom index and to remove any redundant items.

**Table 1:** Symptom indices based on selected items from an 86-item general health questionnaire

General Fatigue Index	Pain Index	Gastrointestinal Index	Sleep Index	Vitality Index
Individual scale items				
Low in energy	Muscle cramps	Abdominal pain	Prolonged Sleep	Libido
Trouble concentrating	Face pain	Diarrhoea	Trouble falling asleep	Night sweats
Muscle weakness	Neck Pain	Constipation	Trouble awakening	Cravings
Forgetfulness	Shoulder pain	Irritable bowel	Disturbed sleep	
Everything an effort	Arm pain	Gastric reflux	Mental fatigue	
Mental confusion	Muscle soreness			
	Heavy limbs			

Eligible subjects were enrolled in stage 2 (*n* = 73) of the program to receive the Fatigue Reviva™ amino acid supplement for a 30 day period. Participants were required to take 20 g of the amino acid based dietary supplement dissolved in 100 ml of water daily. The commercially available nutritional supplement, Fatigue Reviva^TM^ (Top Nutrition Pty Ltd) was comprised of 20 L-amino acids (glycine, proline, glutamine, carnitine, threonine, lysine, alanine, valine, taurine, serine, cysteine, arginine, histidine, isoleucine, phenylalanine, leucine, methionine, glutamic acid, aspartic acid, tyrosine), fructo-oligosaccharide (FOS), malic acid, citric acid, succinic acid, ribose, 13 minerals and 13 vitamins. Production of the supplement Fatigue Reviva^TM^ was carried out according to the restrictions of the NSW Food Regulation 2010 [30], the NSW Food Act 2003 [31] and the Foods Standards Code (FSANZ) [32] whilst the dosages were governed by restrictions of the NSW Food Authority.

At the end of the 30 days, participant data were excluded from the analyses if they had either not taken the supplement for a minimum of 25 days, reported having developed an infection or did not provide a urine sample and completed questionnaires. The data generated from the remaining subjects’ urine samples and questionnaires were included in the analyses. The study was carried out as part of an initial phase of product assessment to determine whether population subgroups exist and if so, does membership of a subgroup influence the efficacy of the supplement. The determination of population subgroups was deemed critical in preparation for future studies in terms of integrating placebo treatments. Participants were also asked to indicate whether they felt the supplement improved their health and whether they would continue to take the supplement if given the opportunity. All participants provided written consent. The trial was registered with the Australian New Zealand Clinical Trials Registry (ACTRN12611000403932) and approved by the University of Newcastle Human Research Ethics Committee (H-2010-1313).

Chalder fatigue scale scores were analysed using Wilcoxon signed-rank test. The general health questionnaire data were assessed by correlation and factor analyses to construct symptom indices appropriate for assessing the impact of amino acid supplementation on general health and wellbeing. Amino acid data were assessed for normality using Shapiro-Wilks W test. Univariate analysis of amino acid data included ANOVA, Wilcoxon matched pairs test and Mann-Whitney *U* test. Multivariate analysis of amino acid data included standard and forward stepwise function analysis of arcsine transformed data. Comparisons were made between the amino acid profiles, general health and levels of fatigue pre- and post-supplementation for a cohort of participants (*n* = 37) who had both successfully completed the amino acid trial and for whom both pre-supplement and post-supplement urine samples were assessed for amino acid content. K-means cluster analysis was used to separate the data collected pre-supplement from 151 participants into three groups based upon their amino acid profiles (relative abundance data). Comparisons were then made between the amino acid profiles pre- and post-supplementation of the three sub-groups based upon the results of the cluster analysis. Statistical analyses were carried out using Dell Statistica (data analysis software system), version 13, software.dell.com, Dell Inc. (2015). Levels of statistical significance were set at *p* < 0.05.

## Results

### Initial evaluation of urinary excretion patterns

**Fig. 2:**
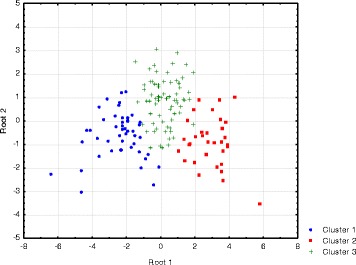
Discriminant function canonical plot of subgroups generated via k-means clustering

Initially, 172 participants (aged 18 years or older) were recruited from the general public for stage 1 of the study. A final study group of 151 subjects provided urine samples and full responses to questionnaires and were reportedly free from any significant medical conditions. The participant group comprised 99 males with an average age of 29.5 ± 11.8 (mean ± SD) and 52 females with an average age of 35 ± 13.7. The first stage of the investigation involved appraising the pre-supplementation urine excretion profiles of the 151 recruits. The amino acid compositions were determined by GC-FID analyses and used to generate a dataset of relative percentage abundances of 29 amino acids and amino acid derivatives. These data were assessed by k-means clustering techniques to determine that the study participants could be partitioned into three clusters based the amino acid relative abundances with a minimum cluster membership of *n* > 10. Standard discriminant function analysis was then applied to determine whether each of the clusters had significantly different amino acid profiles (Wilks’ Lambda = 0.13, F(46,252) = 9.85, *p* < 0.0001) as shown in the canonical plot (Fig. 2) with glycine and histidine levels providing the major discrimination between the groups. The members in each cluster were tightly grouped based upon their urinary excretion profiles and each subgroup was clearly resolved from the other subgroups with minimal overlap.

Seventeen amino acids contributed towards differentiating the composition profiles of the clusters by displaying significant differences in relative abundances for the subgroups and the concentrations of the key components have been summarised in Table 2. Histidine and glycine were the most abundant amino acids in the urinary profiles and displayed different relative abundances for each of the three clusters. Cluster 1 was characterised by a high histidine to glycine ratio of 2.0, cluster 2 displayed a low ratio of 0.4 and cluster 3 had equivalent levels resulting in a ratio of 1.0. Cluster 1 had the highest total urinary concentration of excreted amino acids where histidine levels were 2.4 – 3.6 times those in clusters 3 and 2 respectively. Cluster 1 also had significantly higher concentrations of glutamine, tyrosine and the branch chain amino acids compared with both clusters 2 and 3. In addition, serine and alanine were higher in cluster 1 compared with cluster 3 (Table 2). Cluster 2 was equivalent to cluster 3 in these parameters but was clearly distinguished by the high level of glycine excretion in the urine.

**Table 2:** Characteristics of participant groups by amino acid profile cluster: significant differences in amino acid levels and symptom indices

	Pre-supplement large cohort (*n* = 151)			
Characteristic	Cluster 1 *n = 48*	Cluster 2 *n = 33*	Cluster 3 *n = 70*	*p* value
Mean Age^a^	26.0 (1.2)	37.8 (2.3)	31.9 (1.6)	C1 < C2 = C3 *p* < 0.001
Mean BMI^a^	24.8 (0.4)	24.2 (0.7)	25.4 (0.4)	ns
Proportion Males (number)^a^	75% (36)	30.3% (10)	75.7% (53)	C2 < C1 = C3 *p* < 0.001
Caucasian (%)^a^	95.1%	100%	93.8%	ns
Urine Amino Acids				
Total amino acids μmole/L	6,609 (495)	4,339 (462)	4,425 (306)	C1 > C2 = C3 *p* < 0.01^‡^
Histidine	2,152 (185)	599 (76)	886 (74)	C1 > C2 = C3 *p* < 0.0001
Glycine	1,077 (86)	1,412 (157)	861 (68)	C2 > C3 *p* < 0.001
Glutamine	626 (63)	443 (46)	480 (29)	C1 > C2 = C3 *p* < 0.05
Serine	411 (49)	320 (34)	279 (16)	C1 > C3 *p* < 0.05
Alanine	313 (29)	232 (25)	236 (18)	C1 > C3 *p* < 0.05
Tyrosine	129 (13)	57 (8)	77 (5)	C1 > C2 = C3 *p* < 0.0001
Branched chain amino acids	106 (7)	62 (7)	84 (6)	C1 > C2 = C3 *p* < 0.001
Proline	5.0 (1.9)	9.1 (2.0)	10.7 (1.5)	ns
Symptom Indices				
Chalder total fatigue	12.8 (0.7)	16.0 (1.0)	13.4 (0.8)	C2 > C1 *p* < 0.05
Chalder mental fatigue	4.5 (0.3)	5.9 (0.4)	4.6 (0.3)	C2 > C1 = C3 *p* < 0.05
General fatigue index	3.2 (1.0)	11.2 (1.6)	8.3 (1.1)	C2 > C1 *p* < 0.05
Pain index	5.1 (0.8)	8.1 (1.3)	5.3 (0.7)	ns
Gastrointestinal index	1.7 (0.7)	3.8 (1.0)	2.0 (0.4)	ns
Sleep index	8.2 (1.0)	14.0 (1.9)	11.5 (1.2)	C2 > C1 *p* < 0.05
Vitality index	6.5 (1.2)	9.1 (1.3)	5.5 (0.8)	ns

Statistical tests: ANOVA, Tukey’s HSD for unequal sample sizes, *Chi square; mean (SEM)

The objective partitioning by k-means clustering of subjects based on similarities in their urinary excretion profile removed user-bias in group allocation. Cluster 3 had the highest frequency of membership (46%) followed by cluster 1 (32%) and then cluster 2 (22%) as shown in Table 2. The three subgroups within the study cohort had similar BMI values and were primarily Caucasian. Clusters 1 and 3 had similar mean ages but cluster 2 had a significantly higher mean age than both clusters 1 and 3. Gender was not equally balanced across the three groups where cluster 2 contained predominantly females (*p* < 0.05). Sixteen out of 27 males who identified themselves as athletes were assigned to cluster 1, ten to cluster 3 and only one to cluster 2. There were no significant differences in total amino acid concentrations between the genders in the whole study cohort where males (*n* = 99) had a mean level of 5185 ± 290 μmol/L (mean ± SEM) and females (*n* = 52) 4955 ± 452 μmol/L. Comparisons between the urinary compositions of amino acids in males and females revealed significant differences for glutamic acid (males 16.7 ± 2 vs females 25.0 ± 3 μmol/L, *p* < 0.05), glycine-proline dipeptide (78.0 ± 5 vs 54.7 ± 6 μmol/L, *p* < 0.01), proline-hydroxyproline dipeptide (234.3 ± 15 vs 154.7 ± 19 μmol/L, *p* < 0.01) and tyrosine (96.8 ± 7 vs 72.8 ± 9 μmol/L, *p* < 0.05).

Levels of fatigue were assessed using the Chalder fatigue scale [26] where the average total fatigue score was calculated to be 13.6 ± 5.9 (mean ± SD) for the whole group. However, evaluation of the clusters revealed that cluster 2 had a significantly higher level of total fatigue compared with cluster 1, while clusters 1 and 3 had similar scores (Table 2). The results of the Chalder fatigue scale were mirrored by the fatigue index results derived from the general health questionnaire. Cluster 2 also reported higher levels of symptoms assessed by the sleep index compared with cluster 1.

Correlation analyses were performed to evaluate potential associations between the levels of urinary metabolites and the various symptom indices for each of the clusters. There were distinctive arrays of correlations noted for each of the clusters with some strong positive associations (*r* > .50) noted for cluster 1 females (Table 3). It was evident for females belonging to cluster 1 (*n* = 13) that higher urinary concentrations of glutamic acid, hydroxylysine, methionine, ornithine, proline and valine were all positively correlated with values of the Chalder physical fatigue score (*r* > .55, *p* < 0.05). In the same group, increasing scores in the gastrointestinal index were very strongly associated with higher urinary levels of proline (*r* = .92), methionine (*r* = .82) and valine (*r* = .70). These amino acids were also correlated with a range of other symptoms as shown in Table 3. Females in Cluster 2 only exhibited positive correlations for two amino acids, where the urinary concentration for asparagine was associated with the gastrointestinal index and proline was associated with all four fatigue measures and the gastrointestinal index. In contrast, Cluster 3 females displayed negative associations between general fatigue and alanine, aspartic acid, isoleucine, phenylalanine, and tyrosine. The males in cluster 1 displayed nine negative correlations between amino acids and symptoms, where seven of these involved associations with the gastrointestinal index (Table 4). The males from cluster 2 had positive associations between α-aminobutyric acid and the four fatigue indices. The males from cluster 3 had six symptom indices (all four fatigue measures, pain and vitality) positively associated with β-aminoisobutyric acid and showed no negative correlations.

**Table 3:** Correlations between urinary concentrations of amino acids and symptom index scores for each of the three clusters defined by k-means clustering of urinary amino acid profiles for the female participants

Females: Amino Acid versus Symptom Index	Cluster 1 *n* = 13	Cluster 2 *n* = 21	Cluster 3 *n* = 17
ALA vs General fatigue	ns	ns	*r* = -.56
ASN vs GIT	ns	*r* = 0.50	ns
ASP vs General fatigue	ns	ns	*r* = -.50
GLU vs Chalder total fatigue	*r* = .59	ns	ns
GLU vs Chalder physical fatigue	*r* = .63	ns	ns
HYL vs Chalder physical fatigue	*r* = .57	ns	ns
HYP vs Pain	*r* = .56	ns	ns
ILE vs General fatigue	ns	ns	*r* = -.49
MET vs Pain	*r* = .66	ns	ns
MET vs GIT	*r* = .82	ns	ns
MET vs Sleep	*r* = .59	ns	ns
MET vs Chalder physical fatigue	*r* = .60	ns	ns
ORN vs GIT	*r* = .66	ns	ns
ORN vs Vitality	*r* = .58	ns	ns
ORN vs Chalder total fatigue	*r* = .59	ns	ns
ORN vs Chalder physical fatigue	*r* = .56	ns	ns
PHE vs General fatigue	ns	ns	*r* = -.50
PRO vs General fatigue	*r* = .63	*r* = .48	ns
PRO vs Pain	*r* = .83	ns	ns
PRO vs GIT	*r* = .92	*r* = .45	ns
PRO vs Sleep	*r* = .70	ns	ns
PRO vs Vitality	*r* = .74	ns	ns
PRO vs Chalder total fatigue	*r* = .56	*r* = .52	ns
PRO vs Chalder physical fatigue	*r* = .63	*r* = .49	ns
PRO vs Chalder mental fatigue	ns	*r* = .44	ns
TYR vs General fatigue	ns	ns	*r* = -.49
VAL vs Pain	*r* = .60	ns	ns
VAL vs GIT	*r* = .70	ns	ns
VAL vs Sleep	*r* = .61	ns	ns
VAL vs Chalder total fatigue	*r* = .60	ns	ns
VAL vs Chalder physical fatigue	*r* = .67	ns	ns
Total BCAA vs Chalder physical fatigue	*r* = .59	ns	ns

**Table 4:** Correlations between urinary concentrations of amino acids and symptom index scores for each of the three clusters defined by k-means clustering of urinary amino acid profiles for the male participants

Males: Amino Acid versus Symptom Index	Cluster 1 *n* = 35	Cluster 2 *N* = 21	Cluster 3 *N* = 49
ALA vs GIT	*r* = -.34	ns	ns
ABA vs General fatigue	ns	*r* = .47	ns
ABA vs Chalder total fatigue	ns	*r* = .56	ns
ABA vs Chalder physical fatigue	ns	*r* = .46	ns
ABA vs Chalder mental fatigue	ns	*r* = .60	ns
BAIB vs General fatigue	ns	ns	*r* = .50
BAIB vs Pain	ns	ns	*r* = .37
BAIB vs Vitality	ns	ns	*r* = .38
BAIB vs Total fatigue	ns	ns	*r* = .32
BAIB vs Chalder physical fatigue	ns	ns	*r* = .28
BAIB vs Chalder mental fatigue	ns	ns	*r* = .33
ASN vs General fatigue	*r* = .34	ns	ns
GLY vs GIT	r = -.41	ns	ns
HYP vs Vitality	ns	ns	*r* = .37
LEU vs GIT	*r* = -.49	ns	ns
ORN vs Chalder mental fatigue	ns	ns	*r* = .33
PHE vs GIT	*r* = -.40	ns	ns
PHE vs Chalder physical fatigue	*r* = .35	ns	ns
PHP vs Vitality	*r* = -.37	ns	ns
PHP vs Chalder physical fatigue	*r* = -.38	ns	ns
TYR vs Chalder physical fatigue	*r* = .38	ns	ns
VAL vs GIT	*r* = -.39	ns	ns
Total BCAA vs GIT	*r* = -.47	ns	ns
Total AA vs GIT	*r* = -.36	ns	ns

### Evaluation of amino acid supplementation

Compliance for the supplementation trial was 51% where 37 participants successfully completed the trial by taking the amino acid supplement for a period of 25–30 days as well as returning general health questionnaires and providing post-supplement urine samples. The supplement trial cohort (*n* = 37) comprised 10 females and had an average age of 33.8 years ranging from 19 to 65 years.

Levels of fatigue were assessed using both the Chalder fatigue scale [26] and a general fatigue index developed by the current research team, where lower scores were indicative of a lower level of fatigue for both measures. After completion of the supplement trial, 30 out of the group of 37 participants (81%) reported an improvement in their levels of fatigue. A significant reduction in the mean total fatigue scores for the entire cohort was observed from 13.4 ± 0.8 (mean ± SEM) to 8.8 ± 0.6 (Wilcoxon matched pairs test, *p* < 0.0001) following supplementation. General health and wellbeing was also assessed using an 86 item questionnaire from which several indices were generated (Table 1). Scores were standardised to values out of 40, where a score of “0” indicated that the subject was not affected by any of the symptoms comprising that index, and a score of “40” indicating that the subject experienced all of the symptoms in the index group to a high degree. Following supplementation, significant reductions in questionnaire scores representing potential improvements were seen in the fatigue, sleep and vitality indices (Table 5). In response to the questions regarding their experience using the supplement, 25 responders (68%) indicated that they felt the supplement had improved their health and 28 (76%) stated that they would continue to take the supplement if given the opportunity.

**Table 5:** Reported improvements in general health indices following amino acid supplementation

Symptom index	Pre-supplement Mean (SE)	Post-supplement Mean (SE)	*p* value
Chalder Total Fatigue Chalder Physical fatigue Chalder Mental Fatigue	13.4 (0.8) 8.3 (0.6) 5.1 (0.3)	8.8 (0.6) 5.5 (0.4) 3.3 (0.2)	<0.0001 <0.001 <0.0001
General Fatigue index	6.9 (1.2)	3.7 (1.0)	<0.01
Pain index	4.7 (1.1)	3.9 (1.0)	ns
Gastrointestinal index	2.0 (0.6)	2.7 (0.8)	ns
Sleep index	10.4 (1.5)	6.6 (1.0)	<0.01
Vitality index	6.5 (1.2)	2.2 (0.7)	<0.001

Statistical test: Wilcoxon matched pairs test

The seven participants who did not report any improvements in fatigue were all males and had an average Chalder total fatigue score of 10.0 ± 1.4 (mean ± SEM) prior to supplementation whereas the remainder of the cohort (n = 30) had a significantly higher mean Chalder fatigue score of 14.2 ± 0.8 (Mann-Whitney *U* test, *p* < 0.02). The participants that showed improvements in fatigue thus had a higher level of fatigue prior to commencing the supplementation. The larger group who reported having experienced relief from fatigue ultimately reported post-supplementation fatigue scores that were significantly lower (8.1 ± 0.6, mean ± SEM) than those who did not report improved fatigue (11.7 ± 1.4, *p* < 0.04). Six out of the seven males who did not respond to the amino acid supplementation were classified by their pre-supplementation urinary profile as belonging to cluster 3 and the seventh non-responder was assigned to cluster 1.

## Discussion

Analyses of the urine samples collected from the pre-supplement cohort of 151 healthy participants revealed that it was possible to delineate three clear clusters based upon urinary amino acid profiles. Cluster 3 was the most prevalent of the clusters with its membership totalling 46% of the total cohort with the next largest, cluster 1, comprising 32% of the cohort. An earlier study appraised the amino acid composition of 1993 patient urine samples referred by general practitioners to generate 6 subgroups with a minimum membership of n > 50 [22]. It was thus argued that evidence from this study of the healthy general population and the earlier study of referral patients [22] supported the hypothesis that within the Australian context, phenotypic subgroups exist within the general population based on amino acid excretion characteristics found in urine. It is very likely that with more subjects, a greater number of proportionally smaller subgroups would emerge using k-means clustering techniques. The additional clusters may be relevant to certain lower frequency population phenotypes which could be associated with congenital metabolic anomalies or predispositions to certain medical conditions. Phenotypic membership may also represent epigenetic outcomes where responses by the body to environmental conditions (nutrition, exercise, temperature) or responses to pathogenic challenge which have resulted in alterations in amino acid homeostasis and nitrogen balance.

The objective of measuring the first of the morning fasted urine samples was to minimize confounding from dietary intake in order to assess potential differences in body homeostasis between individuals. Cluster 1 had the highest average concentration of amino acids in the fasted urine samples at approximately 1.5 times the levels measured for clusters 2 and 3. Although total amino acid losses were not determined from 24 h urine samples, the higher first of the morning urinary concentrations suggested that this group may have a greater potential to lose amino acids via urinary excretion suggesting the operation of less efficient mechanisms for kidney reabsorption processes. Histidine was the major component in urine from cluster 1 at 2.4 – 3.6 times the concentrations observed in the other clusters. Histidine has been demonstrated to be an essential amino acid in adults on the basis that histidine deficient diets in both animal models and human studies have led to a negative nitrogen balance with reduced haemoglobin and haematocrit levels [33]. On provision of histidine, the subjects’ nitrogen balances were restored and haemoglobin and haematocrit levels were returned to normal [34]. Haemoglobin protein contains 10% histidine and insufficient supply of this amino acid can lead to anaemia [34]. If a particular human phenotype was more susceptible to histidine loss then there may be a need to compensate for such losses via an increased intake of appropriate protein sources or a negative nitrogen balance would slowly develop with resultant sub-optimal haemoglobin production and associated fatigue. In a previous study carried out by the current research team, amino acid levels were investigated in breast cancer radiotherapy patients who developed fatigue following radiotherapy treatment [12]. Those women who developed significant fatigue had higher levels of urinary histidine excretion prior to treatment compared with those women who remained fatigue free, while their plasma histidine levels dropped below detectable levels following radiotherapy. Cluster 1 from the current study also displayed elevated output of tyrosine, serine and alanine as well as the branch chained amino acids. The excretion of tyrosine in the urine can be indicative of higher non-myofibrillar protein turnover [6, 35, 36] which could further contribute towards fatigue.

Cluster 2 was characterised by glycine as the major excretory product and a predominance of females (70%). Comparison of the male and female urinary amino acid levels for the entire cohort did not reveal any significant differences in the concentrations of histidine, glycine, glutamine, serine or alanine. Glycine is a non-essential amino acid that is primarily derived from serine and is a vital component of human metabolism. Glycine is a major component of collagen (33%) which is the most abundant family of proteins in the body providing structure and form for tissues and organs. Excessive losses of glycine may negatively impact on collagen turnover which has been shown to be higher in females [37]. In addition to protein synthesis it has numerous metabolic functions such the biosynthesis of heme, creatine and nucleic acids [38]. It also has important roles in metabolic regulation, bile secretion and neurological function [38]. As glycine is utilised in so many essential pathways and has high demands in protein synthesis, it has often been referred to as a “conditionally essential” amino acid in reference to situations where *de novo* synthesis can no longer meet demands [38].

A major feature of cluster 3 was a set of positive correlations for the males for β-aminoisobutyric acid (BAIB) with the symptom indices for vitality, pain and all four fatigue measures. This non-protein amino acid exists as two enantiomers, one as a catabolic product of the pyrimidine base thymine found exclusively in urine and the other enantiomer derived from valine is found in plasma [39]. In certain forms of anaemia and in thalassaemia BAIB has been shown to be excreted [40] and has also been negatively associated with haemoglobin levels and erythrocyte count [41]. The vitality index was comprised of the symptoms of night sweats, libido and food cravings, which were grouped together by factor analysis of the symptom profiles. Reduced testosterone levels have been associated with night sweats [42] as well as reduced libido [43] and insulin resistance [44]. Changes in the vitality index in the males may point to reductions in testosterone levels which have previously been associated with fatigue, muscle weakness, reduced muscle protein synthesis and anaemia [44].

The correlation analyses of amino acids with symptom expression measured by the various indices revealed further differences between the three clusters. Although the associations between urinary amino acids and symptoms cannot be interpreted as providing a direct cause and effect mechanism, the data suggested that increased outputs of certain metabolites contributed towards symptom expression in sub-health. The presence or absence of correlations also demonstrated differences between males and females in each of the clusters. Proline for example displayed a number of very strong correlations for females in cluster 1, to a lesser extent in cluster 2 and finally no associations at all in cluster 3. In contrast to the females, there were no associations with proline recorded for males regardless of the cluster membership. Proline together with alanine, glycine, glutamine and valine represented 52% of the amino acid components in resting human plasma [15] which indicates their importance as a circulatory reservoir to meet the body’s demands. The positive associations of urinary proline concentration and symptoms in females relating to fatigue and pain may point to an increased demand in females due to the turnover of non-myofibrillar protein to maintain plasma homeostasis. Proline is the second major component of collagen (after glycine) comprising around 13% of the protein with a further 9% of collagen containing hydroxyproline. The hydroxyproline is formed via post-translational modification of pro-collagen where residues of proline (and lysine) are hydroxylated to form the final collagen product. Hydroxyproline is primarily found in collagen, and the appearance of it in urine can be used as a measure of collagen turnover [45, 46]. It was thus proposed that females from cluster 1 were susceptible to experiencing higher levels of fatigue and pain if they had higher urinary levels of the collagen components hydroxyproline, hydroxylysine and proline. Collagen turnover is generally thought to be higher in females due to the influence of oestrogen on the maintenance of joint flexibility [47]. Chronic fatigue syndrome is reportedly more prevalent in women [48, 49]. The same cluster 1 also had a significantly higher urinary concentration of tyrosine which can also be indicative of an elevated turnover of non-myofibrillar proteins [35, 36]. Higher rates of collagen turnover and losses of proline in urine could therefore have significant implications for meeting the demand for collagen synthesis in a broad range of body tissues and organ systems.

The supplement trial was completed successfully, but only 51% of the recruits were considered to have complied sufficiently by completing a minimum of 25 days of supplementation, completing the questionnaires and providing a final urine sample. Low compliance with trials of this nature is recognised as a common problem [50]. The group that completed the supplementation trial had a similar distribution of gender and cluster membership as observed in the first study survey. The low numbers made it impossible to evaluate differences between the males and females within the subgroups and so the majority of the analyses were focussed on the whole study group with references as appropriate to the incidence of non-responders to the supplement in the various clusters.

The trial group reported experiencing improvements in wellbeing following the supplementation period as evidenced by reductions in the Chalder fatigue scores as well as the general health indices for fatigue, sleep and vitality. The Chalder fatigue scale has been validated for use in the general population as well as those suffering ill health [51]. In the current study, substantial reductions in fatigue were reported with 81% of the cohort indicating an improvement in fatigue and all female participants reporting an improvement in fatigue after taking the supplement. The formulation of the amino acid supplement was such that it would have provided a direct source of amino acids capable of reducing the requirement for non-myofibrillar catabolism and therefore it had the potential to relieve fatigue. The higher levels of urinary histidine (cluster 1) and glycine (cluster 2) could have contributed to limitations in haemoglobin production and collagen turnover respectively and provision of both histidine and glycine in the supplement may have led to enhancements in the synthesis of these proteins [33, 34, 38, 45] and a reduction in associated symptoms such as fatigue and pain.

The seven participants who did not show any improvement following supplementation were all males, six of whom belonged to cluster 3. Cluster 3 had no obvious anomalies of high level excretions of amino acids such as those observed for histidine (cluster 1) and glycine (cluster 2). In addition, the males that did not respond to amino acid support had low Chalder fatigue scores at the beginning of the supplementation period and were therefore not experiencing problematic levels of fatigue in need of treatment. Thus it was concluded that males primarily assigned to cluster 3 via initial urine analysis and who also had low Chalder fatigue scores would be unlikely to achieve improved levels of fatigue from supplementation with amino acids. These subjects would appear to have been free from the symptoms that characterise sub-health. The combined use of the urinary amino acid profiling and the Chalder fatigue scale could be effectively utilised in future testing of amino acid supplementation by allowing selection of those individuals most likely to be experiencing a negative nitrogen balance and fatigue, and who would therefore potentially be more likely to benefit from amino acid support. The use of the general health questionnaire indices provided a framework for appraising the symptoms of sub-health which could be used for monitoring improvements in wellbeing. On this basis, future clinical trials could be developed with appropriate screening for inclusion of significant sub-health issues associated with fatigue, sleep and vitality.

## Conclusions

Using urine excretion profiles of amino acids, it was possible to define three major phenotypic clusters of adult human subjects. Cluster 1 had the highest concentration of amino acids in urine with histidine present at 2.4–3.6 times the concentrations observed in the other clusters while cluster 2 was characterised by a high level of urinary glycine. The three clusters thus had differential excretion characteristics and sets of sub-health symptom profiles, where the females within the three clusters had different associations between nutrient losses and symptom expression compared with the corresponding males. Evidence suggested that collagen/non-myofibrillar muscle protein turnover rates were higher in the females and were associated with the experience of more intense sub-health symptom presentation compared with the males. Provision of the amino acid supplement for 30 days resulted in significant improvements in reported fatigue and sleep for 81% of the trial cohort. It was concluded that people suffering fatigue, assessed as a Chalder fatigue scale score > 13, would be likely to experience significant benefits from amino acid supplementation.

## Abbreviations

AA: Amino acid
ABA: Alpha-aminobutyric acid
ALA: Alanine
ASN: Asparagine
ASP: Aspartic acid
BAIB: Beta-aminoisobutyric acid
BCAA: Branched-chain amino acid
FOS: Fructo-oligosaccharide
GIT: Gastrointestinal tract
GLU: Glutamate
GLY: Glycine
HYL: Hydroxylysine
HYP: Hydroxyproline
ILE: Isoleucine
LEU: Leucine
MET: Methionine
ORN: Ornithine
PHE: Phenylalanine
PHP: Proline-hydroxyproline
PRO: Proline
SEM: Standard error of the mean
TYR: Tyrosine
VAL: Valine
